# Pathological expression of tissue factor confers promising antitumor response to a novel therapeutic antibody SC1 in triple negative breast cancer and pancreatic adenocarcinoma

**DOI:** 10.18632/oncotarget.19175

**Published:** 2017-07-10

**Authors:** Xuesai Zhang, Qingrou Li, Hui Zhao, Lanping Ma, Tao Meng, Jianchang Qian, Rui Jin, Jingkang Shen, Ker Yu

**Affiliations:** ^1^ Department of Pharmacology, Fudan University School of Pharmacy, Shanghai, China; ^2^ Department of Medicinal Chemistry, State Key Laboratory of Drug Research, Shanghai Institute of Materia Medica, Chinese Academy of Sciences, Shanghai, China

**Keywords:** tissue factor, triple negative breast cancer, pancreatic adenocarcinoma, metastasis, antibody-drug conjugate

## Abstract

The pathological presence of tissue factor (TF) in cancer cells promotes tumor-initiated thrombosis and cancer metastasis. We found that TF is aberrantly present in large percentage of aggressive triple negative breast cancer (TNBC) and pancreatic adenocarcinoma (PaC), two most lethal forms of malignancy that urgently need effective treatment. TF expression in TNBC clustered with higher levels of vimentin, basal-type keratins KRT5/14 and caveolin-1 but lower levels of luminal-type biomarkers. We developed a novel and specific anti-TF therapeutic antibody SC1, which displayed an exceedingly high potency against TF extracellular domain (EC_50_: 0.019 nM), TF-positive TNBC- or PaC cells (EC_50_: 2.5 nM), intracellular protease activated receptor 2 (PAR2) signaling (IC_50_: 2-3 nM) and tumor-initiated coagulation (IC_50_: <10 nM). Depletion of TF or SC1-treatment in TNBC or PaC cells inhibited TF-induced cell migration, lung metastasis and tumor growth *in vivo*, accompanied by diminished levels of tumor angiogenesis and stromal fibrosis. We further propose TF as a promising target for antibody-drug conjugate (ADC) development based on its rapid and efficient internalization of SC1-drug conjugate. Both SC1-DM1 and SC1-MMAE elicited exquisite cytotoxicity in TF-positive TNBC and PaC cells (IC_50_: 0.02-0.1 nM) but not in TF-negative cells (>100 nM) achieving >5000 fold target selectivity. Following a weekly intravenous administration, SC1-MMAE and its humanized hSC1-MMAE inhibited TNBC- and PaC tumor growth achieving MED of 0.3-1 mg/kg and were both well tolerated. Thus, the prevalent TF expression in TNBC and PaC renders these challenging tumors highly susceptible to TF-targeted treatment and may offer new opportunity in cancer patients.

## INTRODUCTION

The association between thrombosis and cancer has long been recognized and many clinical and experimental studies have indicated the involvement of hypercoagulopathy in cancer progression and unfavorable outcome [1, 2]. Patients with cancer are often at high risk of developing thrombosis, including venous and arterial thrombosis and systemic syndromes, such as disseminated intravascular coagulation [1–3]. Tissue factor (TF) is a 47 kDa transmembrane glycoprotein that is normally present in subendothelial cells away from circulation; upon tissue damage, TF forms a complex with factor VIIa (FVIIa) to initiate the extrinsic coagulation process. However, it is now known that this tightly controlled hemostatic process can be hijacked by tumors. In this setting aberrant expression of TF in several types of aggressive tumor plays a key role in tumor-initiated thrombosis and is associated with metastatic properties and poor disease prognosis [4–6]. It has been suggested that the tumor TF-induced procoagulant state increases fibrin formation and facilitates tumor cell survival, immune escape and metastasis [7–9]. TF/FVIIa complex also binds and stimulates protease activated receptor 2 (PAR2)-mediated intracellular signaling pathway and activates ERK/MAPK and PI3K pathways, which positively regulates integrin signal, production of chemokines, proangiogenic factors and matrix metalloproteinases thereby facilitating inflammation, angiogenesis and tumor growth [10, 11].

Pathological TF expression has been reported in several cancer types [5, 12–21]. The mechanisms for TF dysregulation involve multiple oncogenic drivers, including those of PI3K, KRAS, EGFRvIII and loss of PTEN or p53 [14–18, 22]. TF is also upregulated in response to VEGF, tumor necrosis factor (TNF)-α and hypoxia-inducible factor-1α (HIF-1α) [15, 22]. In addition, post-transcriptional modification of the TF gene via alternative splicing (asTF) and micro RNA (miRNA) also contribute to tumor development and metastasis [13, 23]. A recent report indicates that TF expression is induced by epithelial-mesenchymal transition (EMT) and triggers a procoagulant state and metastasis of circulating tumor cells [24].

TF represents a potential target for cancer treatment. Numerous experimental studies have provided clues that targeting TF in aggressive tumors will likely be beneficial. Specific blockade of TF functions in tumor cells by TF knockdown, monoclonal antibody (mAb) or by tissue factor pathway inhibitor (TFPI) resulted in inhibition of tumor growth, metastasis and angiogenesis [11, 25–32]. A TF mAb (TF-011)-drug conjugate Tisotumab Vedotin showed promising antitumor activity in preclinical model of TF-positive tumors [33] and has entered patient trial (e.g. NCT02001623).

In this report, we found that TF is frequently expressed in highly invasive triple negative breast cancer (TNBC) and in pancreatic adenocarcinoma (PaC). TNBC and PaC are among the most lethal forms of malignancy in the clinic with extremely poor survival and no effective therapy [34, 35]. To explore the potential for TF as a therapeutic target in these challenging tumor types, we have developed a novel anti-TF therapeutic antibody SC1. Abrogation of TF function in TNBC or PaC cells via ShRNA or TF-mAb SC1 blocked tumor-initiated coagulation process, inhibited TF-PAR2-dependent MAPK/ERK phosphorylation cascade and attenuated tumor growth and metastasis. We also show that the antibody-drug conjugate SC1-ADC exerted excellent antitumor activity in tumor models of TNBC and PaC.

## RESULTS

### TF is aberrantly present in a large percentage of highly invasive Basal-like/TNBC breast cancer and pancreatic cancer

**Figure 1:**
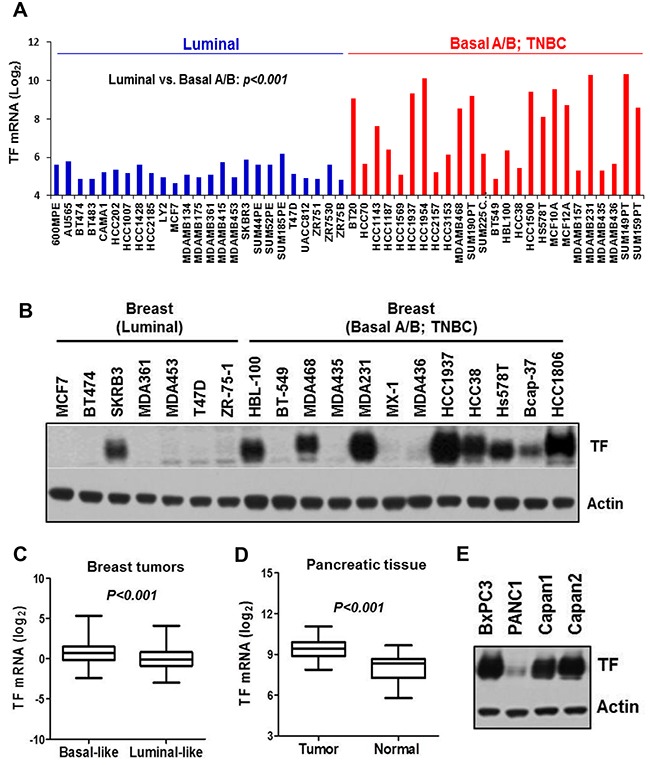
TF is aberrantly expressed in invasive basal-like breast cancer/TNBC and PaC **(A)** TF mRNA levels for 25 luminal- and 25 basal A/B breast cell lines classified by Neve et al. [36] were plotted. **(B)** Total cell lysates prepared from the indicated breast cell lines were immunoblotted for TF protein level. **(C)** Affymetrix array data for 522 breast tumors [37] were sorted into “Basal-like” vs. “Luminal-like” based on mRNA expression ratio (KRT5 plus KRT14)/(KRT8 plus KRT18) [38] with median value of the dataset served as group cutoff. TF mRNA levels are plotted. **(D)** TF mRNA levels in pancreatic tumors vs. normal pancreatic tissues were analyzed from Gene Expression Omnibus (GEO) repository GSE15471 [40]. **(E)** Total cell lysates prepared from the indicated pancreatic cancer cell lines were immunoblotted for TF protein level.

We first analyzed breast cancer cell gene expression profile dataset generated by Neve et al. [36], which included 25 luminal- and 25 basal A/B lines. We found that TF mRNA level was significantly higher in the basal A/B group, many of which are well established TNBC cells (p<0.001) (Figure 1A). Analysis of the Cancer Cell Line Encyclopedia (CCLE) database reached a similar result (not shown). We then examined TF protein in a panel of breast cancer lines by immunoblotting, in which majority of the TNBC lines (MDA-MB-231, HCC1806, Hs578T, HCC1937, HCC38, MDA-MB-468, Bcap-37 and HBL-100) exhibited aberrant TF protein levels compared to that for only one luminal line (Figure 1B). We next examined 522 breast tumor mRNA dataset from TCGA [37]. Tumors were grouped into “Basal-like” or “Luminal-like” based on mRNA expression levels of basal-keratins KRT5/14- vs. luminal-keratins KRT8/18 [38, 39]. We found that TF mRNA level was also higher in the “Basal-like” compared to the “Luminal-like” group of tumors (p<0.001) (Figure 1C). To examine TF levels in PaC, we analyzed GEO dataset with 36 normal- and 36 PaC samples [40]. TF mRNA was much higher in PaC compared to normal pancreas (Figure 1D). This result was verified in PaC cells by immunoblotting (Figure 1E) and was consistent with a previous study employing tissue array [20].

### TF mRNA level in breast cancer correlates with “Basal-like” and “EMT-high” gene signature

**Figure 2:**
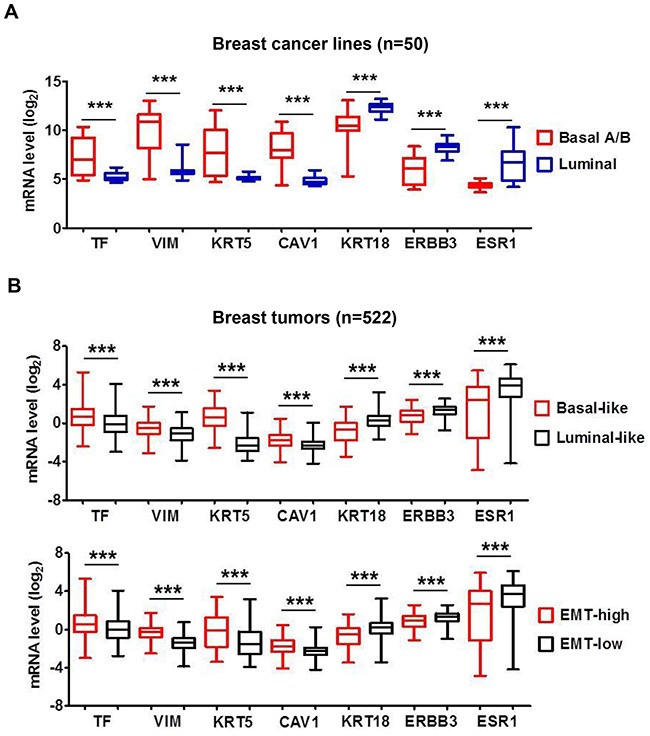
Breast cancer TF mRNA level correlates with “Basal-like” and “EMT-high” signature **(A)** The panel of 50 breast cancer cell lines as in Figure 1A was analyzed employing the Affymetrix array data file (36). Representative mRNA levels of the indicated genes in the 50 cell lines were analyzed and plotted. Vimentin (VIM), keratin-5 (KRT5) and caveolin-1 (CAV1) are representative Basal-markers, while keratin-18 (KRT18), ERBB3 and ESR1 are representative Luminal-markers. **(B)** Affymetrix array data for 522 breast tumors [37] were sorted into “Basal-like” vs. “Luminal-like” or “EMT-high” vs. “EMT-low” based on mRNA expression ratio (KRT5 plus KRT14)/(KRT8 plus KRT18) [38] or vimentin/E-cadherin [39], respectively. Median values of the datasets served as group cutoff. Representative mRNA levels for TF, VIM, KRT5, CAV1, KRT18, ERBB3 and ESR1 were analyzed and compared between “Basal-like” vs. “Luminal-like” tumors (top panel) or “EMT-high” vs. “EMT-low” tumors (bottom panel), respectively. ***, P<0.001.

In the dataset of 50 breast cancer lines TF expression clustered with higher levels of basal-marker vimentin, keratin KRT5/14 and caveolin-1 (CAV1) but lower levels of luminal-marker keratin KRT8/18, ERBB3 and ESR1 (Figure 2A). Comparative analysis of 522 tumors revealed a similar association of TF with higher expression of basal markers but lower expression of luminal markers (Figure 2B top panel). We further grouped tumors by “EMT-high” or “EMT-low” based on levels of vimentin- vs. E-cadherin expression [41]. TF mRNA level was higher in the “EMT-high” tumors (p<0.001), which also clustered with higher basal markers and lower luminal markers (Figure 2B bottom panel). Together, these results indicate that TF is aberrantly present in a large percentage of aggressive tumors with highly invasive and metastatic properties and may present as a promising target for treatment of TNBC.

### SC1 is a novel TF-mAb with high affinity in blocking cell surface TF, TF:FVIIa-PAR2 signaling and tumor-initiated coagulation in TNBC and PaC

**Figure 3:**
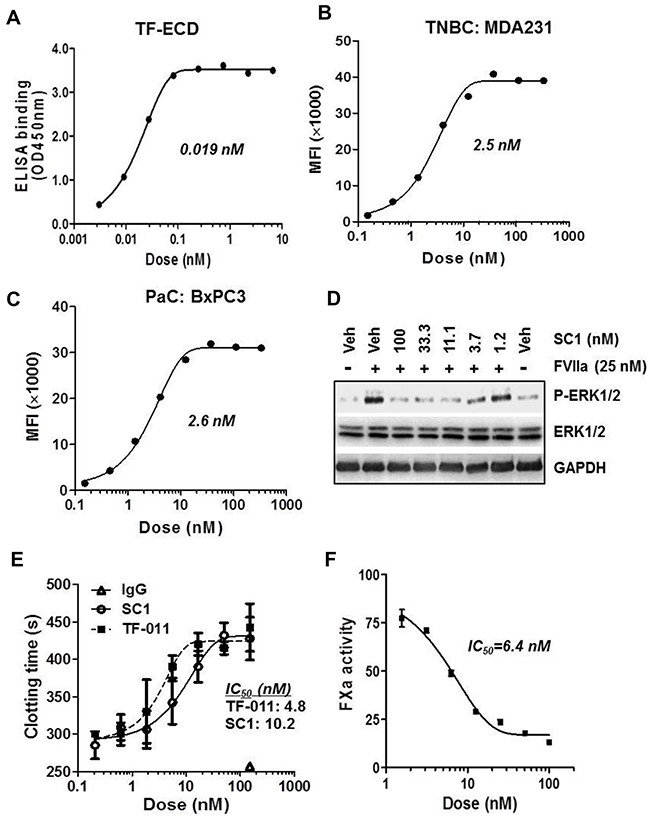
Biochemical and cellular activity profile of SC1 **(A)** Binding affinity of SC1 to TF-ECD as measured by ELISA. SC1 dose response curve and EC_50_ value are shown. **(B and C)** Binding affinity of SC1 to cell surface TF in MDA-MB-231 (B) and BxPC3 (C) was assessed by FACS analysis. SC1 dose response curves and EC_50_ values are shown. **(D)** Serum-starved BxPC3 cells were pre-treated with SC1 for 1 h, stimulated with FVIIa for 15 min and subjected to immunoblotting. **(E and F)** MDA-MB-231 cell-initiated coagulation was inhibited by SC1. Assays for clotting with SC1 and TF-011 (E) and FXa generation with SC1 (F) were performed as described in Methods. Representative assay results are shown. The TF-011 VL/VH regions were prepared as described [49]. MFI, mean fluorescence intensity.

We immunized mice with the extracellular domain of human TF (TF-ECD) and obtained a panel of original human TF-specific monoclonal antibodies (Supplementary Figure 1A). Biochemical affinity assays and cellular pharmacology results identified SC1 (mouse IgG2bκ) as a candidate for further development. IgG variable regions of the SC1 heavy (VH) and light (VL) chains were cloned and sequenced, and sufficient quantity of purified SC1 was obtained (Supplementary Figure 1B). As expected, SC1 immunoblotted well with the cell lysates of high-TF TNBC and PaC (Supplementary Figure 1C) and showed similar immunostaining as the previously established TF-mAb TF-011 [33] in the BxPC3 tumors with the strongest staining on surface of the tumor cells (Supplementary Figure 1D). In ELISA measurement, SC1 binds to TF-ECD with a mean EC_50_ value 0.019 nM (Figure 3A). SC1 elicited a dose-dependent binding to cell surface TF in the TNBC MDA-MB-231 and PaC BxPC3 cells with mean EC_50_ values 2.5 nM and 2.6 nM, respectively (Figure 3B, 3C). In BxPC3 cells, the TF:FVIIa-PAR2-induced ERK/MAPK phosphorylation was blocked by SC1 at 2-3 nM (Figure 3D). Lastly SC1 and TF-011 inhibited both the MDA-MB-231- and BxPC3-initiated coagulation as assessed by clotting time (Figure 3E, Supplementary Figure 2A) or FXa generation assay (Figure 3F, Supplementary Figure 2B) exhibiting the low nM IC_50_ values. These results indicate that SC1 recognizes the functional epitope(s) in TF extracellular domain and perturbs at least two major functions of TF, namely the coagulation function and the TF:FVII-PAR2 intracellular signaling activity.

### SC1 inhibits TF-dependent tumor cell migration *in vitro* and hematogenous metastasis *in vivo*

**Figure 4:**
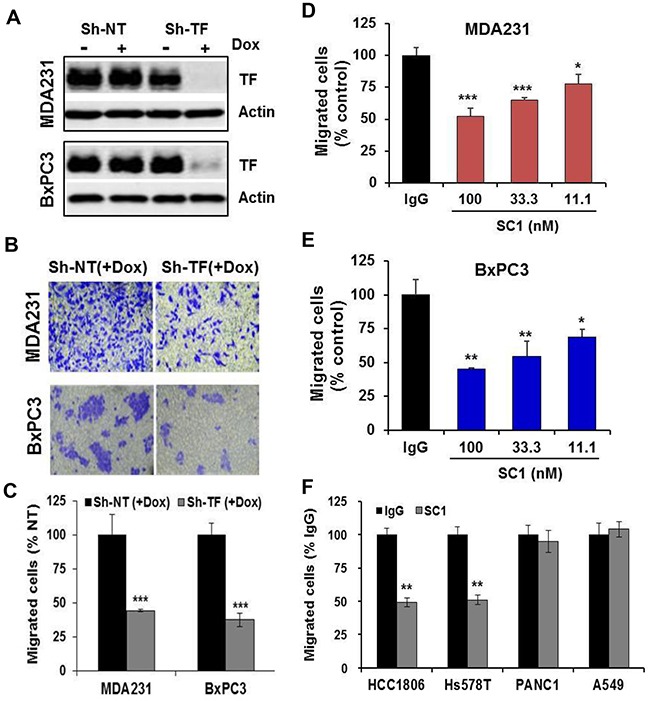
SC1 inhibits TF-dependent tumor cell migration **(A-C)** MDA-MB-231 and BxPC3 cells transfected with TRIPZ non-targeting ShRNA (Sh-NT) or TF ShRNA (Sh-TF) were induced with doxycycline (Dox) for 5-7 days to deplete TF. The cells were subjected to immunoblotting (A) or cell migration assay. Migrated cells were stained with crystal violet (B) and the quantified results are plotted (C). **(D-F)** The indicated cell lines were assayed similarly for cell migration with various doses of SC1 or IgG (D, E) or 100 nM SC1 (F). Quantified results are plotted. *, P<0.05; **, P<0.01; ***, P<0.001.

**Figure 5:**
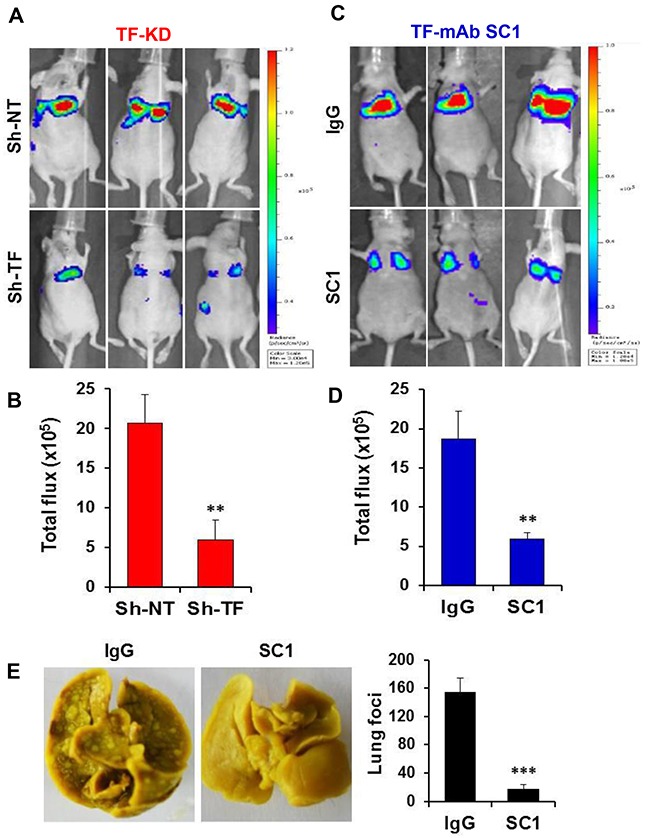
SC1 inhibits TNBC MDA-MB-231 cell lung metastasis **(A and B)** Luciferase-tagged MDA-MB-231 cells expressing TRIPZ Sh-NT or Sh-TF were induced with doxycycline for 7 days and injected into the tail vein of Balb/c nude mice (n=6). Bioluminescence was measured 4 h later as described in Methods (A) and the results quantified based on total photon flux are plotted (B). **(C and D)** MDA-MB-231-Luc cells were mixed with 0.1 mg SC1 or IgG and injected into the tail vein of nude mice (n=4). Bioluminescence was similarly measured and analyzed. **(E)** MDA-MB-231 cells were injected into the tail vein of SCID mice (n=7) and tumor nodules in the lung were examined 6 weeks later (left panel) and quantified (right panel). **, P<0.01; ***, P<0.001.

Given that both the TF:FVIIa-PAR2 signaling [42] and TF-mediated coagulation [43, 44] can contribute to tumor metastasis, we examined the effect of SC1 in anti-tumor cell migration and hematogenous metastasis *in vivo*. We first established the doxycycline (Dox)-inducible TF knockdown cell lines of TNBC MDA-MB-231, HCC1806 and PaC BxPC3, in which efficient depletions of TF were achieved by Dox treatment (Figure 4A, Supplementary Figure 3A, 3C). When TF was depleted, both MDA-MB-231 and BxPC3 cells displayed a significant reduction in cell migration (Figure 4B, 4C). Next, consistent with TF knockdown, treatment with 100, 33.3 and 11.1 nM SC1 resulted in a dose-dependent inhibition of cell migration in the TF-high MDA-MB-231, BxPC3, HCC1806 and Hs578T cells but not in the TF-low PANC1 and A549 cells (Figure 4D, 4E, 4F). For *in vivo* studies, we tagged these cell lines with firefly luciferase gene. Depletion of TF in MDA-MB-231-Luc (Figure 5A, 5B), HCC1806-Luc (Supplementary Figure 3A, 3B) and BxPC3-Luc (Supplementary Figure 3C, 3D) cells all resulted in a profound inhibition in lung invasion as assessed by bioluminescence imaging at 4 h following tail-vein injection of tumor cells. Similarly, co-injection of SC1 with tumor cells also dramatically reduced MDA-MB-231 lung colonization as detected by bioluminescence at 4 h (Figure 5C, 5D) or tumor foci quantified 6 weeks following injection (Figure 5E, 5F). These results strongly indicate that TF plays an important role in tumor cell early metastasis. Blockade of TF function by SC1 and, in addition, the SC1 antibody-dependent cell-mediated cytotoxicity (ADCC) can effectively inhibit this process.

### SC1 attenuates tumor growth *in vivo*

**Figure 6:**
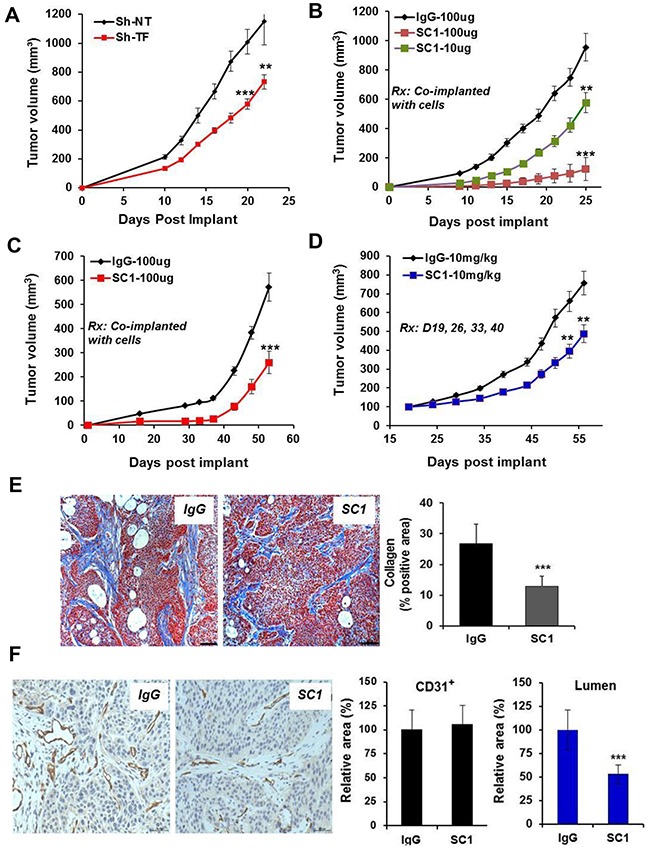
SC1 attenuates tumor growth *in vivo* **(A)** HCC1806 cells expressing TRIPZ Sh-NT or Sh-TF were pre-induced with doxycycline for 7 days and were implanted into the mammary fat pad (MFP) of nude mice (n=10) and maintained with 0.5 mg/mL doxycycline in drinking water. **(B and C)** HCC1806 cells (B) and BxPC3 cells (C) were pre-mixed with the indicated doses of SC1 or IgG and were implanted to MFP (n=10) or flank (n=10), respectively, in nude mice. **(D)** Nude mice bearing established BxPC3 Tumors (n=8) were dosed with IgG or SC1 i.v. 1x weekly as indicated. Tumor growth curves are shown. **(E and F)** On final day of study in C, 3 tumors from each group were collected and subjected to Masson's trichrome staining (E) and CD31 staining (F). Eight view fields per tumor (n=3 tumors) were assessed for quantification analysis. Representative views are shown. Rx, treatment; **, P<0.01; ***, P<0.001.

Although treatment of TF-high tumor cells with SC1 did not acutely block cell proliferation *in vitro* (not shown), TF may be required for optimal tumor growth *in vivo*. Depletion of TF attenuated, albeit moderately, the growth of HCC1806 tumors (p<0.001) (Figure 6A). Co-injection of 10-100 μg SC1 with HCC1806 cells (Figure 6B) or BxPC3 cells (Figure 6C) caused a dose-dependent reduction in tumor growth (p<0.001). Finally, the weekly intravenous administration of SC1 also moderately reduced growth of established BxPC3 tumors (Figure 6D). Next, we examined stromal fibrosis, an important characteristic for PaC progression and therapy impediment [45]. The level of collagen deposition as measured by Masson's trichrome staining was substantially reduced in the SC1-treated BxPC3 tumors compared to that of IgG control (p<0.001) (Figure 6E), indicating an involvement of TF in PaC stromal remodeling. We then analyzed blood vessel distribution by CD31 staining. While SC1 treatment did not reduce the overall level of CD31 staining, it caused a significant reduction in vessel lumen area, indicating a potential role for TF in vessel performance in these tumors (Figure 6F). Together, these results highlight a role for TF in tumor growth, which involves in part its function in tumor stromal remodeling and angiogenesis.

### SC1 antibody-drug conjugates elicit potent cytotoxicity in TF-expressing cancer cells

**Figure 7:**
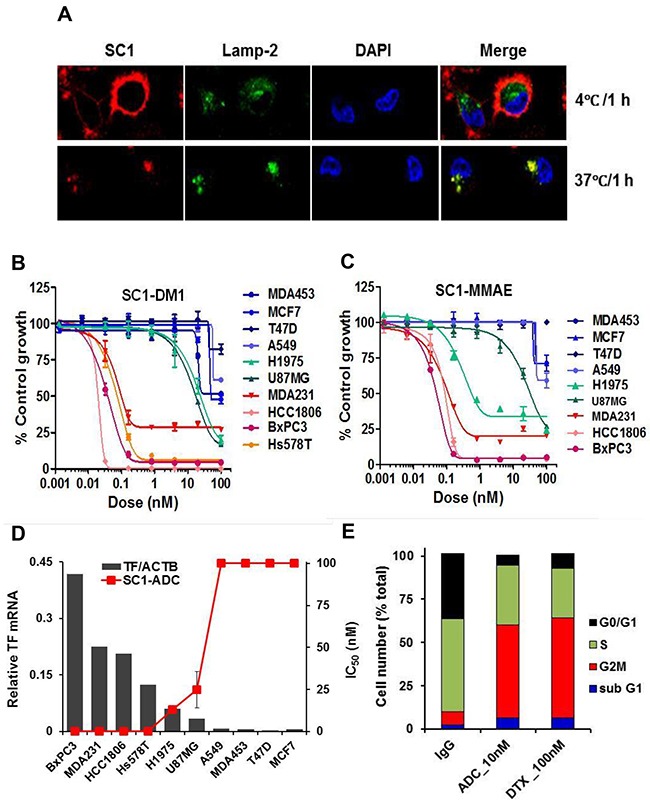
TF-ADCs potently inhibit proliferation of TF-overexpressing tumor cells **(A)** MDA-MB-231 cells were incubated with 10 μg/mL SC1 at 4°C or 37°C for 1 h. Cells were fixed and subjected to immunofluorescence (IF). **(B and C)** A panel of cancer cell lines were treated with various doses of SC1-DM1 (B) or SC1-MMAE (C) for 4 days and analyzed for cell viability. Growth inhibition curves are plotted. **(D)** Growth inhibition IC_50_ values for the cell lines (mean of SC1-DM1 and SC1-MMAE designated as SC1-ADC) were plotted against the TF-mRNA expression levels [36]. **(E)** HCC1806 cells were treated with 10 nM SC1-MMAE or 100 nM docetaxel (DTX) for 48 h. Cell cycle profile as analyzed by FACS are shown. ACTB, β-actin.

To explore the potential utility of SC1 in antibody-drug conjugate (ADC) targeted therapy, we first analyzed its internalization characteristics in MDA-MB-231 cells by confocal microscopy. SC1 was rapidly and extensively internalized into the cytoplasm and co-localized with the lysosomal marker LAMP-2 (Figure 7A) rendering it a suitable candidate for ADC development. We then generated SC1 conjugates with emtansine (SC1-DM1) or monomethyl auristatin E (SC1-MMAE). In a panel of 10 cell lines with high (BxPC3, MDA-MB-231, HCC1806, Hs578T), low (A549, MDA-MB-453, T47D, MCF7) or moderate (H1975 and U87MG) TF expression, both SC1-DM1 and SC1-MMAE elicited exquisite cytotoxicity toward the high-TF cells (IC_50_ ranging 0.02-0.1 nM) but not in the low-TF cells (IC_50_ >100 nM) and were modestly active in the medium-TF cells (IC_50_ 0.55-32 nM) (Figure 7B, 7C, Supplementary Figure 4A, 4C). The IC_50_ values were inversely proportional to TF-mRNA levels, achieving 1000 to >5000 fold target selective cytotoxicity in high-TF TNBC and PaC tumor cells (Figure 7D, Supplementary Figure 4B, 4D) thus validating the potential utility of SC1-ADCs in TF-targeted treatment. Cell cycle analysis showed that treatment of HCC1806 cells with 10 nM SC1-MMAE or 100 nM docetaxel for 48 h resulted in an expected G2/M blockade and cell death in both treatments (Figure 7E). Taken together, SC1-ADCs along with the previously established TF-ADCs [33, 46] represent a new class of highly potent cytotoxic agents for specific targeting of TF-dysregulated cancer cells.

### SC1-MMAE and a humanized hSC1-MMAE demonstrate promising efficacy in preclinical *in vivo* models of TNBC and PaC

**Figure 8:**
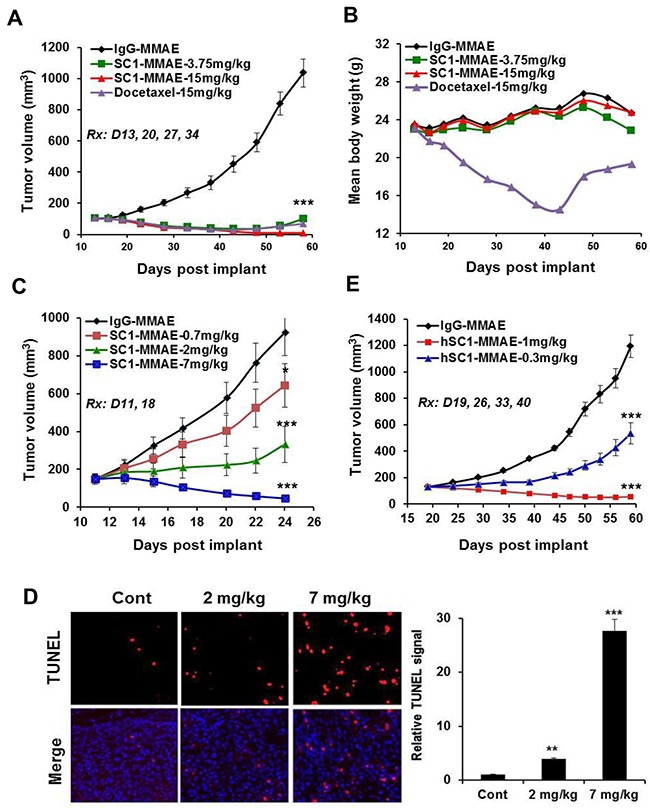
SC1/hSC1-MMAE inhibits TF-expressing TNBC and PaC tumor growth *in vivo* **(A and B)** Mice bearing established BxPC3 tumors (n=8) were treated with SC1-MMAE or docetaxel i.v. 1x weekly as indicated. Tumor growth curves (A) and mouse body weight changes (B) are plotted. **(C)** Mice bearing established HCC1806 tumors (n=8) were treated with the indicated doses of SC1-MMAE. Tumor growth curves are plotted. **(D)** On final day of study in C, HCC1806 tumors were collected 24 h after the last dosing and subjected to TUNEL assay. TUNEL images (n=3) were acquired (left panel) and quantified (right panel). **(E)** Mice bearing established BxPC3 tumors (n=8) were treated with humanized hSC1-MMAE 1x weekly as indicated. Tumor growth curves are shown. *, P<0.05; **, P<0.01; ***, P<0.001.

Nude mice bearing established BxPC3 tumors were treated 1x weekly with IgG-MMAE, SC1-MMAE or docetaxel for 4 weeks. SC1-MMAE elicited a substantial (3.75 mg/kg) or complete tumor regression (15 mg/kg) (Figure 8A). While 15 mg/kg docetaxel showed a similar tumor inhibition as 3.75 mg/kg SC1-MMAE, it caused a severe body weight loss (Figure 8B). Similar treatment of HCC1806-bearing mice with SC1-MMAE for 2 weeks documented a partial to complete tumor inhibition at 0.7 to 2 mg/kg and a nearly complete tumor regression at 7 mg/kg (Figure 8C). TUNEL staining showed that there was a significant increase in apoptosis in HCC1806 tumor tissue in the mice treated with 2 and 7 mg/kg SC1-MMAE (Figure 8D). We developed a humanized SC1 (hSC1; IgG1κ) and its MMAE conjugate (hSC1-MMAE). The unconjugated hSC1 retained full TF-binding affinity *in vitro* (Figure 3A, Supplementary Figure 5A) and *in vivo* antitumor activity compared to SC1 (Figure 6B, Supplementary Figure 5B). 1x weekly treatment with hSC1-MMAE resulted in a substantial inhibition (0.3 mg/kg) or a nearly complete regression (1 mg/kg) of established BxPC3 tumors (Figure 8E). Together these results indicate that SC1/hSC1-MMAE is a potent antitumor agent *in vivo* and can be potentially developed as targeted therapy for treating TF-positive TNBC and PaC.

## DISCUSSION

In analysis of several gene expression profiling database, we found that aberrant TF expression occurs frequently in invasive cancers particularly in “Basal-like” breast cancer, an intrinsic subtype often synonymous with the clinical cohort of TNBC. We also observed TF expression in large percentage of PaC. Because TNBC and PaC are among the most challenging tumors to treat [34, 35], we explored TF-targeting as potential treatment strategy. We have thus far generated a novel and specific TF monoclonal antibody SC1, which displayed exceedingly high inhibition potency against TF extracellular domain, TF-overexpressing TNBC or PaC cells, PAR2 signaling and tumor TF-initiated coagulation. Therefore, SC1 can serve as an excellent pharmacological tool for exploring the therapeutic potential of TF-targeted therapy as cancer treatment.

Previous reports have indicated an association between TF and metastatic property in invasive tumors and have highlighted a critical role for TF-dependent coagulation function in protecting tumor cells in the circulation against anoikis or natural killer cells [8, 24, 43, 44]. We found that TF expression in breast cancer lines and tumors closely clustered with higher levels of EMT (high vimentin/low E-cadherin) and classical basal-type biomarkers KRT5, KRT14 and caveolin-1, while it clustered with lower levels luminal-type biomarkers KRT8, KRT18, ERBB3 and ESR1. In a recent report, EMT was shown to trigger TF expression and metastasis, and co-expression of TF and vimentin was found in subpopulation of circulating tumor cells (CTC) in metastatic breast cancer patients [24]. We have shown that depletion of TF protein or application of SC1 each inhibited TF/FVIIa-induced cell migration in TF-positive TNBC and PaC cells but had no effect in TF-negative cells. These TF-targeted treatments dramatically reduced their ability in metastatic colonization to the lung in mice. Our results are therefore further support a close relationship between TF-triggered procoagulant state and metastatic mechanism in highly invasive tumors including TNBC and PaC.

While depletion of TF or application of SC1 did not directly impact cell proliferation *in vitro*, these treatments significantly attenuated growth of TNBC HCC1806 tumors *in vivo*, indicating that TF is required for optimal *in vivo* growth in these settings. These observations are consistent with the previous TF-depletion study for colon cancer cells [18] and the TF-mAb study for breast cancer cells [11] and collectively highlight the importance of TF in tumor environment. Furthermore, the fact that co-injection of SC1 or hSC1 with TF-positive tumor cells produced better antitumor efficacy compared to that of TF-depletion strongly implies an involvement of the antibody-dependent cell-mediated cytotoxicity (ADCC). Indeed, both the IgG2b (SC1) and IgG1 (hSC1) are well documented for rendering ADCC effect [47], which could present a significant component of the TF-targeted mAb treatment strategy.

In PaC BxPC3 tumor model, treatment with SC1 reduced tumor growth, which was associated with a diminished tumor vessel lumen area leading potentially to a compromised tumor angiogenesis. SC1 may exert such an effect through both the TF-coagulant function and TF-PAR2 signaling [6]. In this context a reduced FXa/thrombin generation indirectly dampens angiogenesis, and the blockade of TF-PAR2 signaling inhibits gene transcription of several key angiogenesis factors such as VEGF, IL-8 and CXCL-1 [10, 11, 28, 29]. The SC1 treatment also reduced tumor stromal fibrosis. Because stromal fibrotic remodeling is a hall mark of PaC and impedes drug penetration [45], targeting TF by SC1 may further improve antitumor efficacy when used in combination with other suitable therapeutic agents. Future studies are required to elucidate the mechanistic detail on how SC1 perturbs the stromal fibrotic response in PaC.

With the FDA approval of Adcetris and Kadcyla, development of ADC as targeted cancer therapy has become a reality and numerous novel ADC candidates have entered clinical trials in cancer patients [48]. Our results support the idea that TF could be a promising target for ADC development. We showed that high levels of TF are expressed in large percentage of invasive tumors particularly in TNBC and PaC, two most lethal forms of malignancy for which new and effective therapies are urgently needed [34, 35]. We observed a rapid (1 h) and extensive SC1 internalization when it binds to TF-positive cancer cells. Both SC1-MMAE and SC1-DM1 elicited a potent cytotoxicity in the TF-positive TNBC and PaC cells *in vitro* but not in TF-negative cells with a selectivity ratio >5000 fold. SC1-MMAE and a humanized hSC1-MMAE were both highly efficacious in *in vivo* models of TNBC and PaC achieving a favorable therapeutic window. It should be noted that because SC1 has poor affinity to murine TF, its tolerability in mice may not translate to humans and requires further evaluation in non-human primates (NHP). Verploegen et al. showed that although TF-011 potently inhibited TF-induced clotting *in vitro*, it did not induce a serious bleeding issue in NHP [49]. TF-011-MMAE Tisotumab Vedotin is well tolerated in humans without major bleeding events and has demonstrated early encouraging antitumor efficacy [50]. Therefore, these results collectively provide both the scientific rationale and clinical evidence in support for TF-targeted ADC as new anticancer treatment.

In summary, our studies have identified a novel and potent anti-TF therapeutic antibody SC1. The prevalence of aberrant TF expression in TNBC and PaC render these two most deadly cancers susceptible to SC1 in preclinical setting and may offer new opportunity in cancer patients.

## MATERIALS AND METHODS

### Chemicals

All general chemicals used in buffers and assays were purchased from Sigma-Aldrich Corporation unless otherwise specified. Specific chemicals are indicated with methods.

### Generation of anti-human TF monoclonal antibody

A histidine-tagged extracellular domain of human TF (TF-ECD) (Sino Biological, Cat#13133-H08H-20) was used to immunize Balb/c mice. Hybridomas were generated by fusion of splenocytes with SP2/0 murine myeloma cells (Sigma-Aldrich). TF-specific hybridomas were identified by screening with TF-specific ELISA as described below. Antibodies with high binding affinity to human TF were scaled up by intraperitoneal injection of hybridomas into Balb/c mice then purified from ascites using protein G affinity chromatography (GE Healthcare). Antibody concentration was determined by A280 measurement and extinction coefficient (A280/E). The variable region sequences of monoclonal antibodies were obtained by GeneRacer kit (Invitrogen, Cat#L1502-02) and DNA sequencing, and were subsequently humanized.

### Cell culture, gene depletion and growth assays

Cell lines of MCF-7, BT-474, SKRB3, MDA-MB-453, MDA-MB-361, MDA-MB-231, HCC1806, BxPC3, Capan-2, A549, H1975 and U87MG were obtained from American Type Culture Collection (ATCC). T47D, ZR-75-1, HBL-100, BT-549, MDA-MB-468, MDA-MB-435, MDA-MB-436, HCC1937, HCC38, Hs578T, MX-1, Bcap-37, PANC-1 and Capan-2 were obtained from the Cell Bank of Chinese Academy of Sciences (CAS, Shanghai). Cells were cultured in a 37°C incubator with 5% CO_2_ using standard cell culture methods and reagents (Invitrogen). To create gene depletion, validated pTRIPZ lentiviral inducible shRNA for TF (Cat#V3LHS_371301, V3LHS_371304) and non-targeting (Cat#RHS4346) were obtained from Open Biosystems. These constructs were packaged in 293T cells and validated according to manufacturer's instruction. Virus-infected tumor cells were selected by a pre-determined concentration of puromycin. TF depletion was induced by incubating the cells with 1 μg/mL of doxycycline (Dox) for 5-7 days. Cell growth assays were conducted in 96-well plate at 8-10% starting confluence with treatment for 4 days. Cell viability was assessed using MTS reagent (Promega) as reported previously [51].

### Assays of TF antibody binding and internalization

Antigen binding was assayed via ELISA. ELISA plate was coated with 0.5 μg/mL histidine-tagged TF-ECD for overnight, sequentially washed, blocked with 2% BSA in PBS and incubated with various doses of TF antibody, then detected with HRP-labeled goat anti-mouse IgG (Jackson ImmunoResearch, Cat#115-035-146). To measure binding to cell surface TF, MDA-MB-231 or BxPC3 cells (3×10^5^) were incubated with various doses of TF antibody in 200 μL serum-free medium at 4°C for 1 h, washed with PBS and bound antibody was detected with R-PE-conjugated goat anti-mouse IgG (Jackson ImmunoResearch, Cat#115-116-071) by FACS analysis (BD FACSArialTM IIU).

To analyze the internalization of antibody into cancer cells, MDA-MB-231 cells were plated at 50% confluence in glass bottom cell culture dish (NEST, Cat#801002) and incubated with 10 μg/mL of TF antibody at 37°C or 4°C for the indicated time. The cells were fixed with 4% formaldehyde, permeablized and detected with Alexa Fluor 647-conjugated AffiniPure donkey anti-mouse IgG (Jackson ImmunoResearch, Cat#715-605-150). The lysosomes were visualized via incubating cells with a rabbit anti-human LAMP-2 (Abcam, Cat#ab125068) and detected with Alexa Fluor 488-conjugated AffiniPure donkey anti-Rabbit IgG (Jackson ImmunoResearch, Cat#711-545-152). Images were acquired under confocal microscope (Zeiss, LSM710).

### Coagulation assays

Coagulation assay was performed as described [26]. Briefly, MDA-MB-231 or BxPC3 cells (30,000 in 50 μL) prepared in HBSS buffer with 5 mM CaCl_2_ were mixed with various doses of TF antibody (50 μL) at 25°C for 15 min on a Titer Plate Shaker. The clotting was initiated by the addition of 50 μL citrated human plasma and read immediately for opacity at 405 nm on a kinetic plate reader every 15 sec up to 2 h. Clotting time was plotted as a function of antibody concentration. For FXa assay, 15,000 MDA-MB-231 or BxPC3 cells were incubated with 3 nM FVIIa (Novo Nordisk) and TF antibody (100 μL) at 25°C for 20 min. Reaction was initiated by adding 50 μL FX (final 50 nM) (Haematologic Technologies, Cat#HCX-0050) for 10 min and terminated with 25 μL of 1 M EDTA. FXa was measured by adding 25 μL of 3 mM S2765 (Aglyco, Cat#AG00-0102) and read immediately on a kinetic plate reader every 15 sec up to 1 h.

### Protein lysates and immunoblotting

After various treatments cells were lysed using NuPAGE-LDS sample buffer (Invitrogen). For profile of cancer cell line panel, cells were lyased as described [52]. Total cell lysates were immunoblotted with antibodies for human TF (R&D Systems, Cat#MAB2339), P-ERK and ERK (Cell Signaling), GAPDH and actin (Bioworld).

### Cell migration and experimental metastasis

For cell migration, appropriate number of cells in 200 μL serum-free medium were added to the upper chamber of a Transwell system (Corning, Cat#3422) with TF antibody or control IgG and allowed to migrate for 8 h at 37°C, 5% CO_2_ toward serum-containing medium in the lower chamber. Cells on the underside of the membrane were stained with crystal violet and counted under a microscope in 5 representative viewing fields at 200x magnification. For lung metastasis, 6-8 weeks old female Balb/c nude mice or SCID mice were purchased from B&K Laboratory Animal Corporation. Luciferase-tagged MDA-MB-231 cells (2×10^6^ cells in 200 μL PBS) were mixed with TF antibody or control IgG and injected into the lateral tail vein of mice. For bioluminescence imaging 4 h after cell injection, mice were given D-luciferin potassium salt (150 mg/kg) intraperitoneally and imaged 6 min later for 1 min exposure in an IVIS Spectrum *in vivo* imaging system. Mice were sacrificed 6 weeks after cell injection and lung tumor nodules were counted.

### Antibody-drug conjugates

Anti-TF IgG was conjugated with monomethyl auristatin E through a protease-cleavable valinecitrulline (VC) dipeptide or with maytansine by N-succinimidyl 4-(N-maleimidomethyl) cyclohexane-1-carboxylate (SMCC) [53]. The average drug-antibody ratio (DAR) was 3.6.

### *In vivo* tumor growth, immunohistochemistry (IHC) and apoptosis

*In vivo* efficacy studies were performed under protocols approved by institutional IACUC of Fudan University. Xenograft tumor models were established in female Balb/c nude mice by mammary fat pat (MFP)- or subcutaneous implantation with 3×10^6^ cells (HCC1806) or 10×10^6^ cells (BxPC3), respectively. For co-injection experiments, cells were mixed with TF-mAb or control IgG (n=10) immediately before injection. For therapeutic treatments, established tumors were staged at 100-200 mm^3^ (n=8), various doses of mAb or ADC were administered i.v. 1x weekly following the indicated schedules. Tumor growth was monitored twice weekly and calculated using the formula V=LW^2^/2 (where V=volume, L=length and W=width). For IHC, tumors were snap-frozen in liquid nitrogen or formalin fixed for 24 h and embedded in paraffin. Slides were processed for staining with anti-CD31 (Cell Signaling) or Masson's trichrome (Sigma, Cat#HT15-1KT). For apoptosis assay, tumor slides were processed for terminal deoxynuleotidyl transferase dUTP nick end labeling (TUNEL) using an assay kit (KeyGen Biotech, KGA7061) following vendor's instruction manual. Images were acquired using Leica microscope (model DMI4000D). Staining levels were analyzed on the basis of mean optical density quantified by software image pro plus 6.0.

### Statistical analysis

All numerical data were presented as mean ± standard deviation (SD) except for mouse xenograft studies data which were presented as mean ± standard error (SE). Numerical data processing and statistical analysis were performed with Microsoft Excel and GraphPad Prism 5 software. P values were calculated using unpaired two-tailed Student-t test. In all tests, differences with P values < 0.05 were considered to be statistically significant.

## SUPPLEMENTARY MATERIALS FIGURES AND TABLES


