# Supplementary material for: Pathological expression of tissue factor confers promising antitumor response to a novel therapeutic antibody SC1 in triple negative breast cancer and pancreatic adenocarcinoma
Source: Oncotarget. 2017 Jul 10;8(35):59086–102. doi: 10.18632/oncotarget.19175 (PMC5601716; doi:10.18632/oncotarget.19175)
Supplement: Supplementary file 1 [file oncotarget-08-59086-s001.pdf]

Pathological expression of tissue factor confers promising antitumor response to a novel therapeutic antibody SC1 in triple negative breast cancer and pancreatic adenocarcinoma

SUPPLEMENTARY MATERIALS

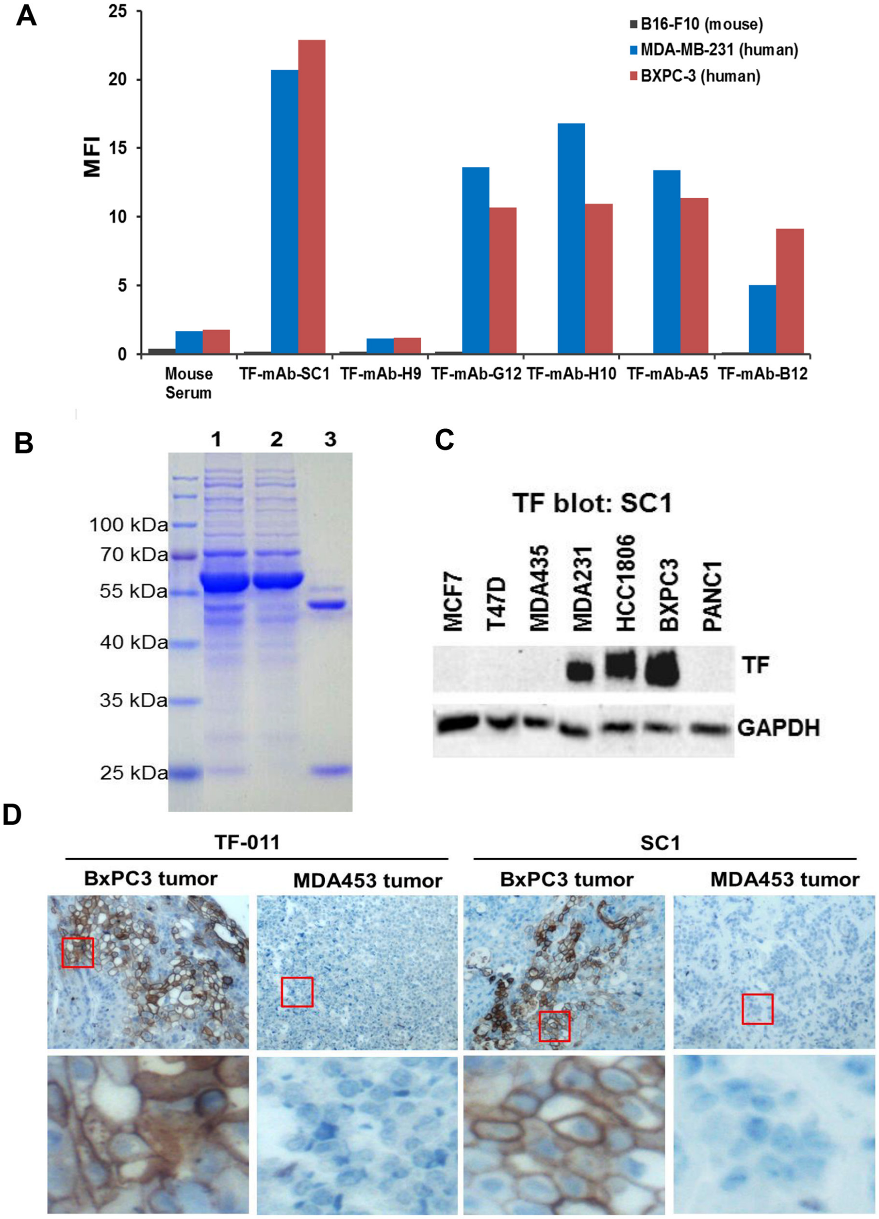

Supplementary Figure 1: Identification and cloning of a novel anti-TF mAb SC1. (A) A panel of originally identified TF mAbs were prepared from mouse ascites and assayed for binding to cell surface human TF in MDA-MB-231 and BxPC3 cells or mouse TF in B16-F10 cells by FACS analysis. The B16-F10 has been verified to express mouse TF (not shown). The binding activities (mean fluorescence intensity, MFI) at a constant 10 µg/mL are plotted. **(B)** SDS-PAGE was run to show the ascites (lane-1), Protein-G column flow-through (lane-2) and the purified TF-mAb SC1 (lane-3). **(C)** The indicated TF-positive and TF-negative cell lines were immunoblotted with SC1. **(D)** Nude mouse xenograft tumors of BxPC3 (TF-high) or MDA-MB-453 (TF-low) were collected and subjected to immunostainings with 2 µg/mL TF-011 or SC1 as described in Method. TF-011 and SC1 show very similar staining pattern with the strongest binding to surface of tumor cells.

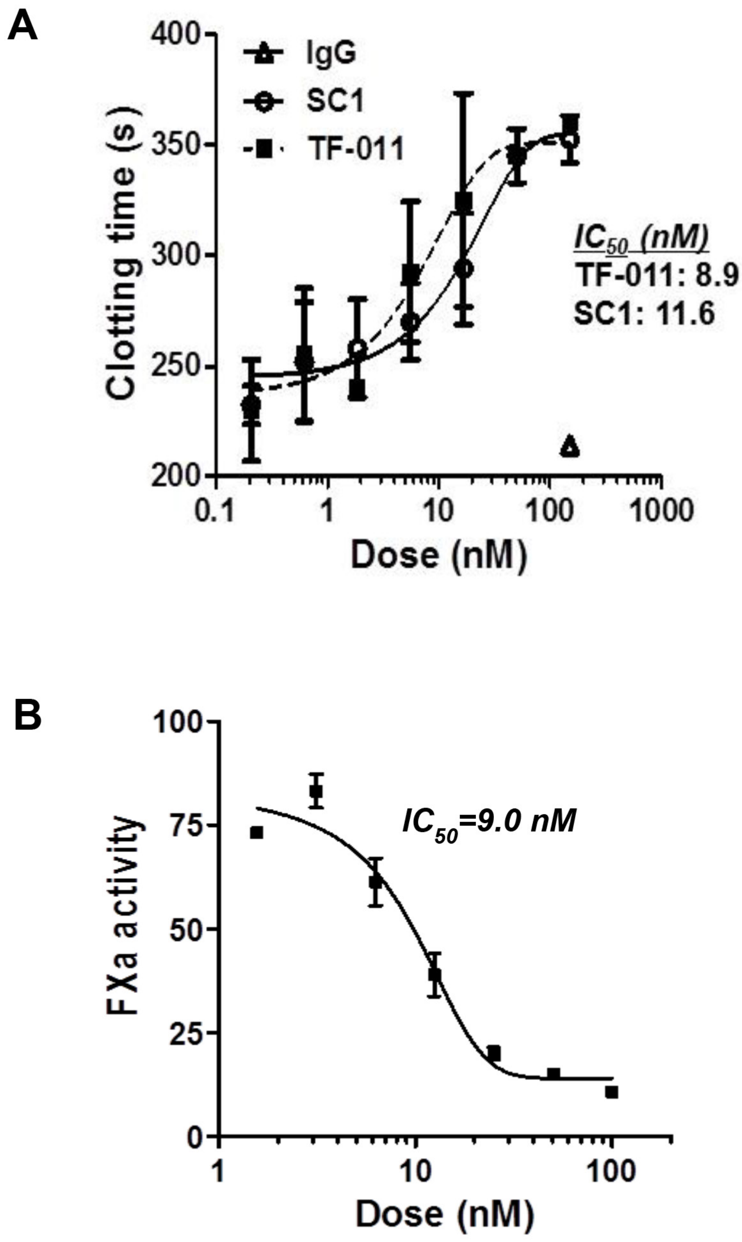

Supplementary Figure 2: SC1 and TF-011 inhibit tumor-TF-induced coagulation. The inhibition assays for clotting (**A**) and FXa generation (**B**) were performed with BxPC3 cells similarly as in Figure 3E, 3F. The TF-011 VL/VH regions were prepared as described [49].

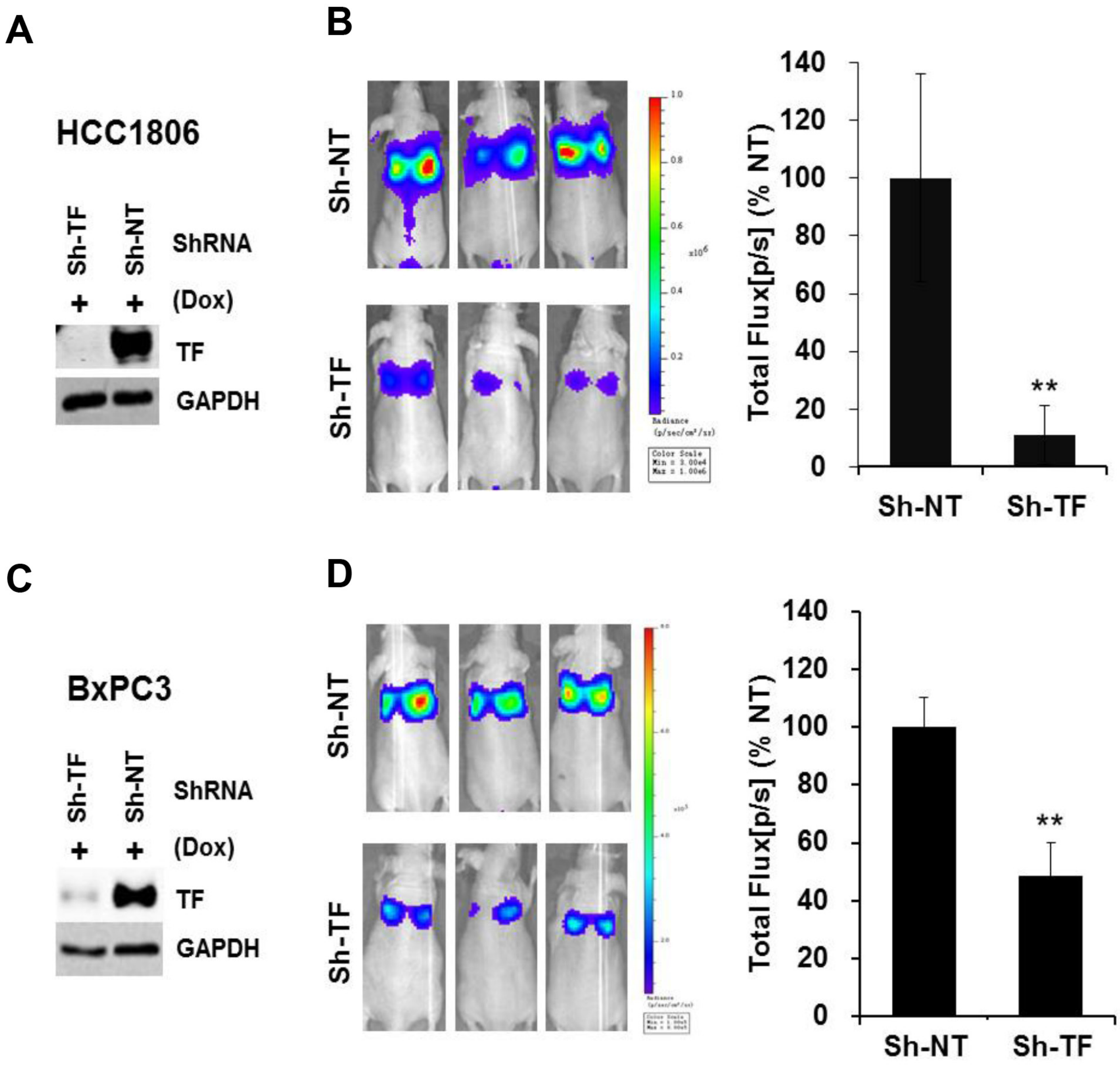

Supplementary Figure 3: Depletion of TF in TNBC HCC1806 and PaC BxPC3 cells reduces lung metastasis capacity. **(A)** Luciferase-tagged HCC1806 cells expressing the indicated pTRIPZ-ShRNAs were induced with doxycycline for 7 days and subjected to immunoblotting. **(B)** Cells as in (A) were injected into the tail vein of Balb/c nude mice (n=4). Bioluminescence was measured 4 h later (left panel) and quantified (right panel). **(C)** Luciferase-tagged BxPC3 cells expressing the indicated pTRIPZ-ShRNAs were similarly induced for TF depletion. **(D)** Cell as in (C) were injected into the tail vein of Balb/c nude mice (n=4). Bioluminescence was measured 4 h later (left panel) and quantified (right panel). **, P<0.01.

A

SC1-MMAE	
Cell line	IC_{50} (nM)
MDA453	>100
MCF7	>100
T47D	>100
A549	>100
H1975	0.549±0.065
U87MG	32.491±5.362
MDA231	0.112±0.014
HCC1806	0.088±0.01
BxPC3	0.049±0.003

B

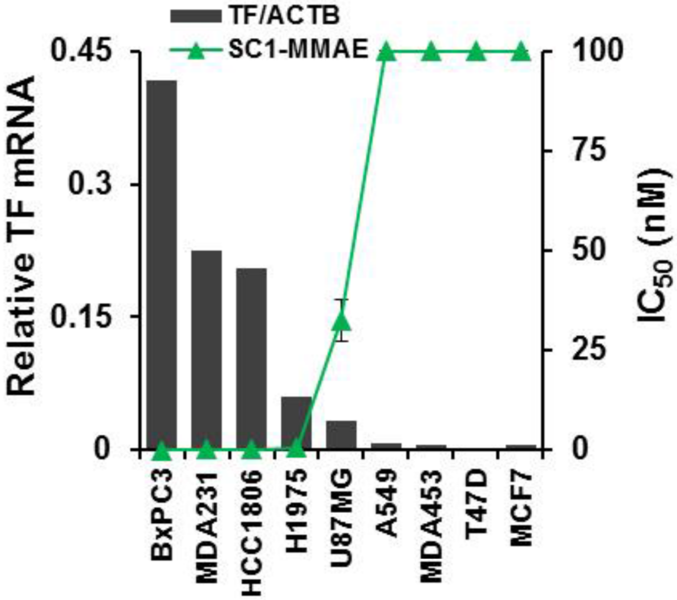

C

SC1-DM1	
Cell line	IC_{50} (nM)
MDA453	>100
MCF7	100
T47D	>100
A549	>100
H1975	25.395±4.482
U87MG	17.203±0.928
MDA231	0.098±0.025
HCC1806	0.022±0.006
BxPC3	0.038±0.005
Hs578T	0.074±0.009

D

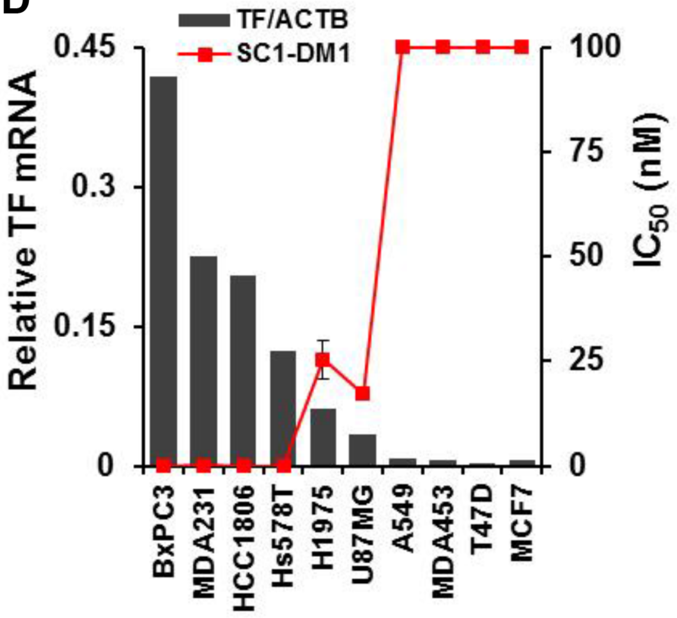

Supplementary Figure 4: SC1-ADCs show potent and specific cytotoxicity in TF-overexpressing tumor cells. (A) The indicated cell lines were treated with various doses of SC1-MMAE for 4 days and IC_{50} values are shown. **(B)** Co-plot of SC1-MMAE IC_{50} with relative TF-mRNA expression level (36). **(C)** The same set of cell lines were treated with SC1-DM1 for 4 days and IC_{50} values are shown. **(D)** Co-plot of SC1-DM1 IC_{50} with TF-mRNA level as in B.

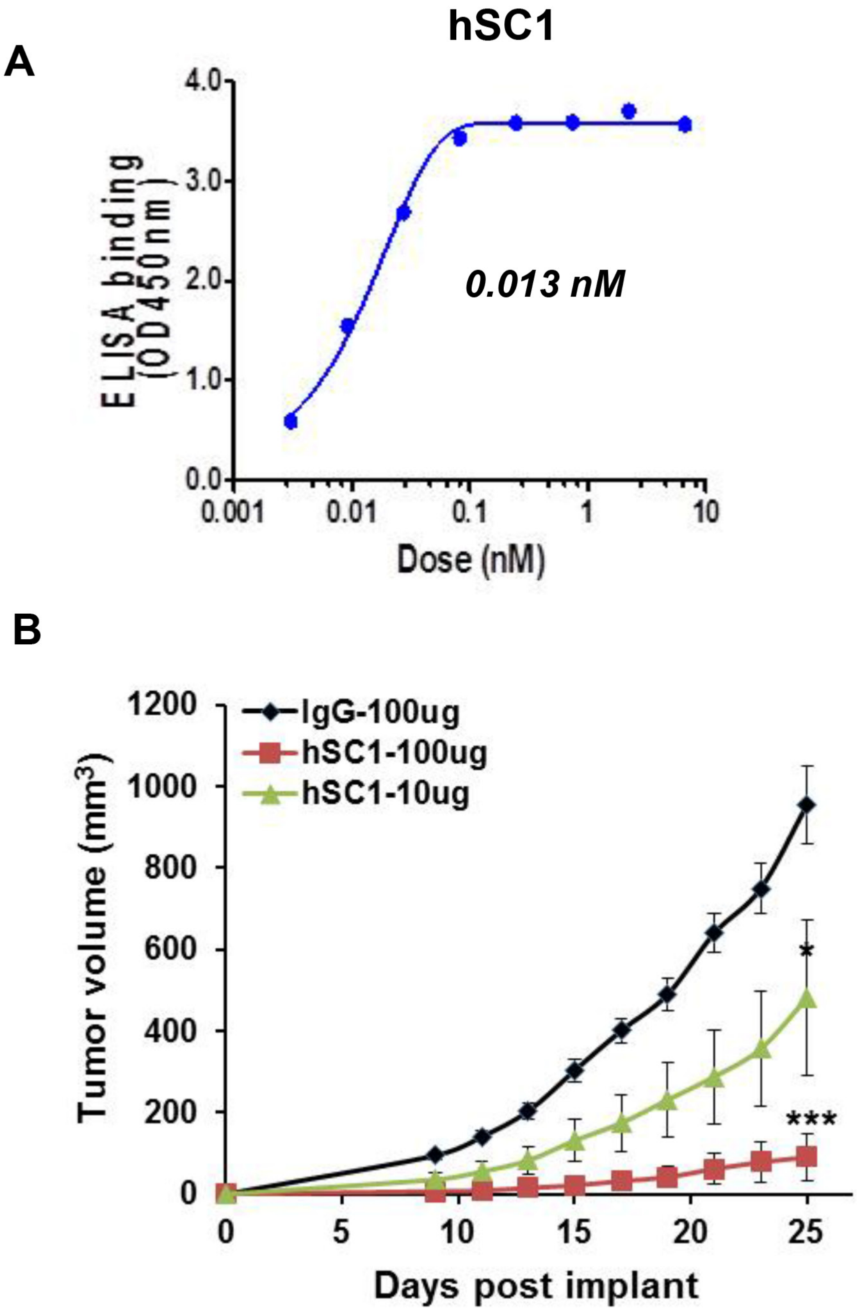

Supplementary Figure 5: Binding affinity profile of the humanized SC1 (hSC1). **(A)** Binding activity of hSC1 to TF-ECD was assessed by ELISA with EC_{50} value indicated. **(B)** HCC1806 cells were pre-mixed with the indicated doses of hSC1 or IgG and were implanted into MFP (n=10) in nude mice. Tumor growth curves are shown. *, P<0.05; ***, P<0.001.
